# Genome Analysis of Multidrug-Resistant *Shewanella algae* Isolated From Human Soft Tissue Sample

**DOI:** 10.3389/fphar.2018.00419

**Published:** 2018-04-26

**Authors:** Yao-Ting Huang, Yu-Yu Tang, Jan-Fang Cheng, Zong-Yen Wu, Yan-Chiao Mao, Po-Yu Liu

**Affiliations:** ^1^Department of Computer Science and Information Engineering, National Chung Cheng University, Chia-Yi, Taiwan; ^2^Department of Energy, Joint Genome Institute, Walnut Creek, CA, United States; ^3^Department of Veterinary Medicine, National Chung Hsing University, Taichung, Taiwan; ^4^Division of Clinical Toxicology, Department of Emergency Medicine, Taichung Veterans General Hospital, Taichung, Taiwan; ^5^School of Medicine, National Defense Medical Center, Taipei, Taiwan; ^6^Department of Nursing, Shu-Zen Junior College of Medicine and Management, Kaohsiung City, Taiwan; ^7^Rong Hsing Research Center for Translational Medicine, National Chung Hsing University, Taichung, Taiwan; ^8^Division of Infectious Diseases, Department of Internal Medicine, Taichung Veterans General Hospital, Taichung, Taiwan

**Keywords:** *Shewanella algae*, wound infection, snake bite, virulence, whole-genome sequencing, colistin resistance, carbapenem resistance

## Introduction

*Shewanella algae* is a gram negative, facultative anaerobe, which was first isolated from red algae (Simidu et al., 1990). With its natural habitat being an aquatic environment, it has been rarely reported as a human pathogen (Khashe and Janda, 1998). *S. algae* infections, however, have become increasingly common over the past decade (Janda, 2014). Its clinical presentations include soft tissue infections, blood stream infections and biliary tract infections (Janda and Abbott, 2014), which cause considerable morbidity and mortality (Liu et al., 2013). These situations are now being more challenging for clinicians and public health sectors to deal with in view of worsening antimicrobial resistance (Yousfi et al., 2017).

The advance in genomic technologies has changed many fields of healthcare, particularly in bacterial genomics (Punina et al., 2015; Chaudhry et al., 2016). Such technology detects potential resistance determinants and virulence repertoires, depicts phylogenic relationship among microorganisms and indirectly sheds light on candidate antimicrobials (Kim et al., 2017). Facing the challenge of emerging bacterial infection, obtaining high-quality genomic data has become more and more important (Patrignani et al., 2014). So far, nonetheless, there are few genetic information regarding virulence and resistance *S. algae* and genomic resources for studying this bacterium are limited.

To fulfill this gap, we conducted a genomic analysis of multidrug-resistant *S. algae* YHL. The strain isolated from human wound infection was sequenced on high-throughput sequencing platform, which was followed by assembly, annotation and final comparative analysis with computational tools and database. Virulence and resistance genes of *S. algae* YHL strain are identified. The genomic information will serve as the basis of further investigation of *S. algae* and development of antimicrobial strategies.

## Materials and methods

### Strain isolation and antimicrobial susceptibility tests

Strain YHL was isolated from a wound sample collected 2 h after bitten by a Chinese cobra (*Naja atra*). Written informed consent was obtained from the patient for publication of this case and was approved by the Institutional Review Board of the Taichung Veterans General Hospital (C10257). The tissue culture yielded gram-negative bacilli and was preliminarily identified as *S. algae* (designated YHL) through the use of 16S rRNA gene sequence analysis (Weisburg et al., 1991). The primers used for amplification of the 16S rRNA gene were B27F (5′-AGAGTTTGATCCTGGCTCAG-3′) and U1492R (5′-GGTTACCTTGTTACGACTT-3′). The PCR product was then sequenced and compared with the bacterial 16S rRNA gene sequences in the GenBank database of the National Center for Biotechnology Information using the BLASTn (optimized for Megablast) algorithm (Camacho et al., 2009).

Antimicrobial susceptibility testing and interpretation were conducted using the automated Vitek 2 system, according to the manufacturer's instructions. The antibiotics were used for this study include ampicillin/sulbactam, piperacillin/tazobactam, cefazolin, ceftriaxone, ceftazidime, cefoperazone, flomoxef, cefepime, imipenem, gentamicin, amikacin, trimethoprim/sulfamethoxazole, ciprofloxacin, tigecycline, and colistin.

### DNA extraction, library preparation, genome sequencing

*S. algae* YHL was grown at 37 °C on trypticase soy broth (Becton, Dickinson, Franklin Lakes, NJ) (Satomi et al., 2006). Cell was harvested and the genomic DNA was extracted from cells collected in exponential growth phase with the QIAGEN Genomic-tip 100/G kit and Genomic DNA Buffer Set (QIAGEN, Valencia, CA) based on the manufacturer's instructions (Thór et al., 1995; Syn and Swarup, 2000). DNA concentrations were quantified using the Qubit dsDNA HS Assay kit with the Qubit 2.0 fluorometer (Life Technologies). A total of 2 μg of DNA sample was sheared using a Covaris S2 device (Covaris Inc.) (Rohland and Reich, 2012). Sheared DNA was used to build indexed PCR-free libraries through the use of a multiplexed high-throughput sequencing TruSeq DNA Sample Preparation Kit (Illumina, San Diego, CA) according to the manufacturer's protocols after minor modifications (van Dijk et al., 2014). Sequencing was performed on an Illumina MiSeq platform (Loman et al., 2012). The whole genome sequencing was performed with a read length of 250 bp paired-end reads on the Illumina MiSeq sequencing platform and generated 4,142,984 reads. The total read depth was 257-fold coverage, with a mean read length of 301 bp.

### Genome assembly and annotation

The reads were filtered using duk (http://duk.sourceforge.net/), quality trimmed with the FASTQX-toolkit fastqTrimmer to remove low quality reads (https://github.com/agordon/fastx_toolkit). Sequencing data was first assembled using Velvet v. 1.2.07 (Zerbino and Birney, 2008), and the resulted contigs were then scaffolded with ALLPATHS v. R46652 (Butler et al., 2008).

The annotation of the strain YHL was performed using the National Center for Biotechnology Information (NCBI) Prokaryotic Genomes Automatic Annotation Pipeline (PGAAP). Functional classification of these annotated genes was carried out by RPSBLAST v. 2.2.15 (Altschul et al., 1990) in conjunction with COGs (Clusters of Orthologous Groups of proteins) databases (*e* < 0.001). The expect value (*e*-value) in BLAST describes the number of alignment hits expected to be seen by chance when searching a sequence database, which is analogous to the *p*-value in statistical hypothesis testing.

### Whole-genome average nucleotide identity analysis

To measure the overall genome relatedness between YHL and the other *Shewanella* genomes, the Average Nucleotide Identity (ANI) (Konstantinidis and Tiedje, 2005) was calculated based on a modified algorithm proposed by Lee et al. (2016). An ANI value of 95% was set as the cut-off for species demarcation.

### Identification of pan-genome core genes and strain-specific genes

The protein-coding genes of YHL were compared with those in *S. algae* MARS 14, *S. algae* JCM 21037, *S. algae* C6G3, *S. algae* BrY, and *S. algae* CSB04KR (Supplementary Table S1). Specifically, the protein sequences of all strains were BLAST-aligned with each other. A gene is considered to be present in both strains if their alignment identity is at least 90% and the alignment coverage is at least 90%. These two cutoffs of 90% were determined by the statistics of alignment and coverage of all gene-pairs in the *S. algae* strains. We observed 90% to be a good cutoff for balancing sensitivity and specificity. We consider each gene to be strain-specific if it is only presented in the strain and lost in all other strains. On the other hand, the genes presented in all strains are the pan-genomic core genes.

### Phylogeny analysis

Seven published *Shewanella* strains were obtained from the NCBI database (Supplementary Table S2). Strains YHL and MARS 14 are human isolates, while JCM 21037, C6G3, BrY, and CSB04KR, and JCM14758 are environmental isolates. We reconstructed the phylogeny separately using 16s rRNA, gryB, and whole-genome sequences. The sequences of 16s rRNA and gryB were extracted from their genomes, aligned against each other using MEGA7, and used for inferring phylogeny (Kumar et al., 2016). Whole-genome phylogeny analysis was carried out by use of the REALPHY pipeline (Bertels et al., 2014). The remaining genomes were aligned against each other using bowtie2 in order to construct multiple sequence alignments (Langmead and Salzberg, 2012). Single Nucleotide Polymorphisms (SNPs) and short insertions and deletions (indels) within the multiple sequence alignments were extracted for subsequent phylogeny reconstruction. Finally, MEGA7 was again used to infer their phylogeny with 1,000 bootstraps.

### Mapping of virulent factors

The potential virulent genes in the *S. algae* YHL genome were identified using the Virulence Factor Database (VFDB) (Chen et al., 2016). The protein sequences of annotated genes are first aligned against VFDB protein sequences of a full dataset (Set B), using BLASTX under the following criteria: alignment coverage (for both query and subject) is at least 50%, and there is an *e* < 1e-5. If multiple virulent genes are overlapped at the same locus in the genome, only the best-aligned virulent factor gene is retained.

### Annotation of antibiotic-resistance genes

The *S. algae* YHL resistome is annotated through using the Resistance Gene Identifier (RGI) from the Comprehensive Antibiotic Resistance Database (McArthur et al., 2013), along with the Integrated Microbial Genomes (IMG) database (Markowitz et al., 2012). The RGI prediction of resistome is based on homology and SNP models, where strict criteria were chosen for prediction. In homolog models, BLAST is used to detect functional homologs with the antimicrobial resistant genes. In contrast, SNP models identify candidate genes which acquire mutations conferring antimicrobial resistant genes based on curated SNP matrices. The *S. algae* YHL resistome is predicted through aligning it against the IMG database using BLASTN with a 95% sequence identity threshold.

## Results and discussion

### General genome features of *S. algae* YHL

The final assembled genome consisted of 27 scaffolds (>2 kbp) with a total size equal to 4,850,439 bp, with a mean GC content of 52.96% (Supplementary Figure S1). The maximum contig size was equal to 976,090 bp, and the N50 size equal to 357,371 bp. The gene annotation included 4,276 protein Coding Sequences (CDSs), 85 tRNA genes and 13 rRNA gene. No extrachromosomal elements were detected in YHL.

### Identification of *Shewanella algae* core genes and strain-specific genes

**Figure 1:**
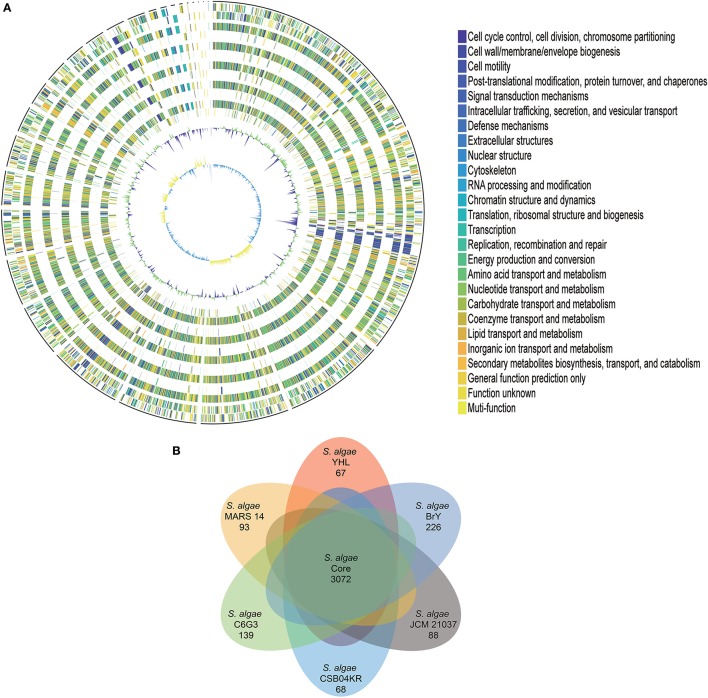
Gene orthology analyses between *S. algae* YHL, *S. algae* MARS 14, *S. algae* JCM 21037, *S. algae* C6G3, *S. algae* BrY, *S. algae* CSB04KR. **(A)** Circles show from the outermost to the innermost: 1. DNA coordinates. 2,3 Function-based color-coded mapping of the CDSs predicted on the forward and reverse strands of the *S. algae* YHL genome, respectively. 4. Orthologous CDSs shared between *S. algae* YHL and *S. algae* BrY. 5. *S. algae* YHL-specific CDSs, compared with *S. algae* BrY. 6. Orthologous CDSs shared between *S. algae* YHL and *S. algae* C6G3. 7. *S. algae* YHL-specific CDSs, compared with C6G3. 8. Orthologous CDSs shared between *S. algae* YHL and *S. algae* CSB04KR. 9. *S. algae* YHL-specific CDSs, compared with *S. algae* CSB04KR. 10. Orthologous CDSs shared between *S. algae* YHL and *S. algae* JCM 21037. 11. *S. algae* YHL-specific CDSs, compared with *S. algae* JCM 21037. 12. Orthologous CDSs shared between *S. algae* YHL and *S. algae* MARS-14. 13. *S. algae* YHL-specific CDSs, compared with *S. algae* MARS 14. 14. GC plot depicting regions above and below average in green and violet, respectively. 15. GC skew showing regions above and below average in yellow and light blue, respectively. **(B)** Illustration showing the number of CDSs shared between the six strains. Core (blue) and strain-specific (skyblue) genome size of *S. algae*.

The protein-coding genes of *S. algae* YHL were compared to human isolate *S. algae* MARS 14, along with environmentally-associated *S. algae* JCM 21037, *S. algae* C6G3, *S. algae* BrY, and *S. algae* CSB04KR, in order to identify the orthologous core genes shared across all strains and strain-specific genes. Figure 1A depicts both the positions and color-coded functions of *S. algae* YHL genes in comparison with all other strains, whereas the numbers of orthologous and strain-specific genes are shown in Figure 1B. In summary, the pan-genome of *S. algae* consisted of 3,072 core genes shared across all strains, whereas 67 genes are specific to *S. algae* YHL. Functional analysis of YHL-specific genes revealed that, in addition to hypothetical proteins, a relative abundance of the gene is involved in replication and repair, along with cell wall/membrane/envelop biogenesis (Supplementary Figure S2).

### Investigation of *S. algae* YHL-specific virulent genes

To better understand the pathogenic potential of YHL, we further investigated whether any of these 67 YHL-specific genes are well-known virulent factors by BLAST search against the VFDB. The analyses revealed that both *neuA* and *lpsB* are unique virulent genes found only in the *S. algae* YHL strain (Supplementary Table S3). *neuA* encoding bifunctional O-acetylesterase/sialic acid synthetase and is essential in sialic acid biosynthesis. Studies have shown that sialic acid-containing capsules in pathogenic bacteria restrict host immune activation (Bouchet et al., 2003) and have been recommended as a therapeutic target (Ourth and Bachinski, 1987). The other YHL-specific virulent gene, *lpsB*, encodes a glycosyltransferase that is essential in lipopolysaccharide synthesis and is an important virulent determinant in many pathogenic Gram-negative bacteria (Parsons et al., 1988; Wang et al., 2014).

In order to investigate the distribution of common virulent genes on a pan-genome scale, we annotated and compared all the virulent genes in the six *S. algae* strains. Most virulent genes are commonly shared across all strains, and these core virulent genes are related to metalloprotease, flagella, capsular polysaccharide biosynthesis, T2SS (Type 2 secretion system) and T6SS (Type 6 secretion system), heme biosynthesis and outer membrane heme receptors. We further identified homologs of gene involved the mannose-sensitive hemagglutinin (MSHA) type IV pilus (*mshABCD*), which is involved in adhesion to mucosal receptors and formation of biofilms (Heidelberg et al., 2002). Gene related to endothelial adhesion was also detected and could be potential drug targets (Trenti et al., 2017).

### High-resolution phylogeny revealed by whole-genome sequences

OrthoANI analysis revealed that *S. algae* YHL were identical to MAR14, JCM 21037, C6G3, and CSB04KR in terms of nucleotide sequences, sharing an ANI > 98% (Supplementary Figure S3). The *S. algae* YHL was almost identical to the human pathogenic *S. algae* MAR14, yet distinct (< 75%) from other *Shewanella* species.

**Figure 2:**
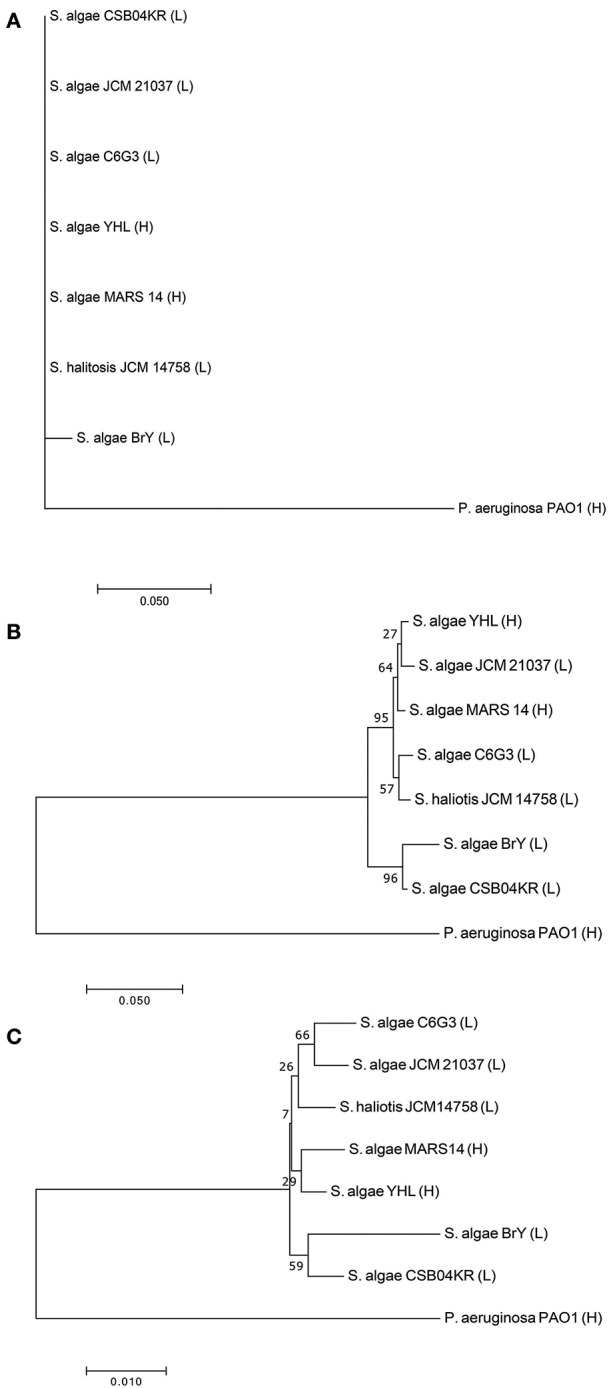
Phylogeny of high- and low-pathogenic *Shewanella* strains. **(A)** Phylogenetic tree constructed with 16S rRNA gene sequences of *Shewanella* strains. **(B)** Phylogenetic tree constructed with gyrB gene sequences of *Shewanella* strains. **(C)** Phylogenetic tree constructed with the whole-genome sequences of *Shewanella* strains. Note that the ending abbreviation H stands for high-pathogenic human isolates, whereas L represents low-pathogenic environmental isolates.

Seven *Shewanella* strains (and one outgroup, *P. aeruginosa*) were downloaded from the public database (see Method, Supplementary Tables S1, S2). Two strains (YHL and MARS 14) are high-pathogenic, while the remaining are low-pathogenic (i.e., JCM 21037, C6G3, BrY, CSB04KR, and JCM14758). We compared the phylogeny, separately reconstructed using 16s rRNA, *gryB*, and whole-genome sequences (Figure 2). Phylogeny based on 16s rRNA had no resolution amongst these closely-related strains (Figure 2A). Although we used *gryB*, a housekeeping gene with higher mutation rates which provides a relatively higher resolution, it was still insufficient to reliably distinguish high-pathogenic (i.e., YHL and MARS 14) from the other five low-pathogenic strains (Figure 2B). Finally, the phylogeny reconstructed using whole-genome sequences separated the high-pathogenic *S. algae* strains from the other low-pathogenic strains (Figure 2C). Together, these results support the importance of whole-genome sequences for high-resolution reconstruction of phylogeny, and for measuring the degree of pathogenicity in *Shewanella* strains.

### Understanding of *S. algae* YHL multidrug resistance via resistome analysis

The *S. algae* YHL strain is found to be multidrug-resistant, including colistin (MIC of 8 μg/ml), imipenem (MIC of 16 μg/ml), ampicillin and cefazolin (Supplementary Table S4). To explore the possible genetic factors leading to this multidrug resistance, antibiotic-resistant genes (ARGs) in the YHL genome was annotated using the CARD and IMG (see Method, Supplementary Table S5). Functional analysis of these ARGs revealed that they may contribute resistance to β-lactams (*bla*_OXA−55_, *bla*_MUS−1_) and polymycins (*pmrA, pmrB, pmrC, pmrE, pmrF*). In addition, the mutant porin gene (*omp36*) was also detected.

Currently, there is no large scale study on the antimicrobial resistant profiles of *S. algae*. Case reports and case series' have shown that *S. algae* are typically susceptible to carbapenems, extended-spectrum cephalosporins, aminoglycosides, fluoroquinolones, and trimethoprim-sulfamethoxazole, while also being resistant to colistin (Janda, 2014; Janda and Abbott, 2014). However, the emergence of carbapenem resistance in *S*. *algae* has been reported in Korea (Kim et al., 2006; Byun et al., 2017), France (Cimmino et al., 2016), and India (Srinivas et al., 2015). The mechanism of carbapenem resistance in *S. algae* is proposed to be associated with the presence of *bla*_OXA−55_ (Yousfi et al., 2017). However, OXA-55 β-lactamase displays low hydrolytic activity against carbapenems and is also present in carbapenem-susceptible *S. algae* (Walther-Rasmussen and Hoiby, 2006). The resistance to carbapenems in *S. algae* may be the result of a combined action involving OXA-55 β-lactamase and a secondary resistance mechanism.

Structural changes in porin can lead to carbapenem resistance, particularly in the presence of β-lactamases. Reports have demonstrated the correlations between carbapenem-resistance with both porin changes and oxacillinases (Uz Zaman et al., 2014). The deficiency of *omp36* impairs the diffusion of carbapenem and plays a major role in the development of carbapenem resistance (Pages et al., 2008; Catel-Ferreira et al., 2012). Moreover, porin and β-lactamase are strongly synergistic. An altered porin phenotype is also commonly associated with the expression of degradative enzymes, such as β-lactamases, which efficiently confer a high level of β-lactam resistance (Nikaido, 1989).

The most common mechanism of resistance to colistin is modification of lipopolysaccharides with Phosphoethanolamine (PEtN) and 4-amino-4-deoxy-L-arabinose (L-Ara4N) mediated by PhoP/PhoQ and PmrA/PmrB two-component systems (Olaitan et al., 2014). A gene expression study of *S. algae* suggested *pmrC* (*eptA*) playing a prominent role in polymyxin resistance (Telke and Rolain, 2015). A genetic analysis in our study of putative candidate target genes associated with polymyxin resistance found in YHL (*pmrA, pmrB, pmrC, pmrE, pmrF, phoP*, and *phoQ* genes) further revealed that the *pmrA, phoP*, and *phoQ* genes harbor substitutions at positions that confer resistance to polymyxin. We speculate that these mutations could likely play a role in colistin resistance exhibited by *S. algae* YHL.

Here we present the first genomic study of carbapenem- and colistin-resistant *S. algae* from snake bite wound. Our data provides basic information regarding further resistance, along with virulent studies of *S. algae* infections, thus enabling for more precise anti-infective therapy in the future. In addition, large scale genome surveillance for this unique pathogen should be instituted to provide more detailed information about its pathogenesis and treatment.

## Data access

This genome sequence of *Shewanella algae* YHL has been deposited in GenBank under accession number LVDU01000000, BioProject PRJNA312015.

## Author contributions

Y-TH, J-FC, and P-YL designed and coordinated the study and carried the data analysis. Y-YT and Y-TH performed the bioinformatics analysis. Z-YW and Y-CM carried out the experiments and interpreted data for the work. Y-TH, Y-YT, and P-YL wrote manuscript. Y-TH, J-FC, and P-YL checked and edited the manuscript. All authors have read and approved the manuscript.

### Conflict of interest statement

The authors declare that the research was conducted in the absence of any commercial or financial relationships that could be construed as a potential conflict of interest. The reviewer LB and handling Editor declared their shared affiliation.
