# Supplementary material for: Genome Analysis of Multidrug-Resistant Shewanella algae Isolated From Human Soft Tissue Sample
Source: Front Pharmacol. 2018 Apr 26;9:419. doi: 10.3389/fphar.2018.00419 (PMC5932639; doi:10.3389/fphar.2018.00419)
Supplement: Supplementary file 1 [file Table_1.DOCX]

| **Supplementary Table S1.** Summary of the *S.algae* strains in this study |  |
| --- | --- |
| \| Strain \| Accession numbers \| Source \| Host \| Country of origin \| Reference \| \| --- \| --- \| --- \| --- \| --- \| --- \| \| YHL \| NZ_LVDU00000000 \| wound \| Human \| Taiwan \| this study \| \| MARS 14 \| NZ_CDQH00000000 \| Lung \| Human \| France \| [1] \| \| JCM 21037 \| NZ_BALO00000000 \| surface of red algae \| algae \| Japan \| NA \| \| C6G3 \| NZ_JPMA00000000 \| marine sediment \| intertidal sediment \| France \| [2] \| \| BrY \| NZ_MDKA00000000 \| Great Bay Estuary \| water \| United States \| [3] \| \| CSB04KR \| NZ_MBFW00000000 \| Gut \| Apostichopus japonicus \| Korea \| [4] \| | |

[1]

Cimmino T, Olaitan AO, et al. 2015. Whole genome sequence to decipher the resistome of Shewanella algae, a multidrug-resistant bacterium responsible for pneumonia, Marseille, France. Expert Review of Anti-infective Therapy. 142:269-275

[2]

Aigle A, Bonin P, et al. 2017. Physiological and transcriptional approaches reveal connection between nitrogen and manganese cycles in Shewanella algae C6G3. Scientific Reports 7:44725

[3]

Turick CE, Caccavo F Jr, et al. 2008. Pyomelanin is produced by Shewanella algae BrY and affected by exogenous iron. Canadian Journal of Microbiology. 544: 334-339.

[4]

Hong HH, Choi H, et al. 2017. Genome Sequences of Two Shewanella spp. Isolated from the Gut of the Sea Cucumber Apostichopus japonicus (Selenka, 1867). Genome Announc. 529: e00674-17
