# Supplementary material for: Genome Analysis of Multidrug-Resistant Shewanella algae Isolated From Human Soft Tissue Sample
Source: Front Pharmacol. 2018 Apr 26;9:419. doi: 10.3389/fphar.2018.00419 (PMC5932639; doi:10.3389/fphar.2018.00419)
Supplement: Supplementary file 2 [file Table_2.DOCX]

**Supplementary Table S2.** Genome features of *S.algae* strain YHL, MARS 14, JCM 21037 , C6G3, BrY, CSB04KR

| Strain | Assembly level | size (Mb) | GC content (%) | Gene | Protein | rRNA | tRNA | Pseudogene | Release Date | Last Modify Date |
| --- | --- | --- | --- | --- | --- | --- | --- | --- | --- | --- |
| YHL | Scaffold | 4.85 | 53.00 | 4,316 | 4,141 | 13 | 86 | 71 | 2017/09/20 | 2017/09/22 |
| MARS 14 | Scaffold | 5.01 | 52.90 | 4,467 | 4,257 | 12 | 104 | 90 | 2015/01/29 | 2017/04/10 |
| JCM 21037 | Contig | 4.83 | 53.00 | 4,435 | 3,407 | 4 | 64 | 956 | 2014/04/01 | 2017/04/09 |
| C6G3 | Contig | 4.88 | 53.10 | 4,608 | 3,153 | 25 | 85 | 1,340 | 2015/03/19 | 2017/04/10 |
| BrY | Contig | 4.55 | 52.40 | 4,062 | 3,936 | 0 | 46 | 69 | 2016/11/07 | 2017/04/13 |
| CSB04KR | Scaffold | 4.80 | 53.10 | 4,305 | 4,140 | 10 | 91 | 59 | 2016/11/01 | 2017/04/13 |
