# Supplementary material for: Genome Analysis of Multidrug-Resistant Shewanella algae Isolated From Human Soft Tissue Sample
Source: Front Pharmacol. 2018 Apr 26;9:419. doi: 10.3389/fphar.2018.00419 (PMC5932639; doi:10.3389/fphar.2018.00419)
Supplement: Supplementary file 4 [file Table_4.docx]

**Supplementary Table S4. Antimicrobial susceptibility proﬁles of the *S. algle* YHL**

| **Antibiotic(s)** | **MIC (μg/ml)^a^** | **Susceptibility^b,c^** |
| --- | --- | --- |
| Penicillins |  |  |
|  |  |  |
| Ampicillin/sulbactam | ≤ 2 | S |
| Piperacillin/tazobactam | ≤ 16/4 | S |
|  |  |  |
| Cephalosporins |  |  |
| Cefazolin | ≥ 64 | R |
| Ceftriaxone | ≤ 0.25 | S |
| Ceftazidime | 0.5 | S |
| Cefoperazone | ≤ 4 | S |
| Flomoxef | ≤ 2 | S |
| Cefepime | ≤ 0.12 | S |
|  |  |  |
| Carbapenems |  |  |
| Imipenem | 16 | R |
|  |  |  |
| Aminoglycosides |  |  |
| Gentamicin | ≤ 1 | S |
| Amikacin | ≤ 2 | S |
|  |  |  |
| Fluoroquinolones |  |  |
| Ciprofloxacin | ≤ 0.25 | S |
|  |  |  |
| Trimethoprim/Sulfamethoxazole | ≤ 20 | S |
|  |  |  |
| Tigecycline | ≤ 0.5 | S |
|  |  |  |
| Polymyxins |  |  |
| Colistin | > 16 | R |

**^a^** Breakpoint testing only.

**^b^** R, resistant; S, susceptible.

**^c^** Susceptible interpretation based on CLSI M100-S27 guidelines for Other Non-Enterobacteriaceae, except for ampicillin/sulbactam, cefazolin, cefoxitin, and colistin, where the U.S. Food and Drug Administration breakpoints were applied.
