# Supplementary material for: Genome Analysis of Multidrug-Resistant Shewanella algae Isolated From Human Soft Tissue Sample
Source: Front Pharmacol. 2018 Apr 26;9:419. doi: 10.3389/fphar.2018.00419 (PMC5932639; doi:10.3389/fphar.2018.00419)

Supplementary Figure S1. Circular genome map of *S.algae* YHL. The circles show from the outermost to the innermost: 1. DNA coordinates; 2, 3. Function-based color coded mapping of the CDSs predicted on the forward and reverse strands. Various functions are assigned different colors; 4. tRNA genes; 5. rRNA genes; 6. GC plot with regions above and below average in green and violet, respectively; 7. GC skew showing regions above and below average in yellow and light blue, respectively.

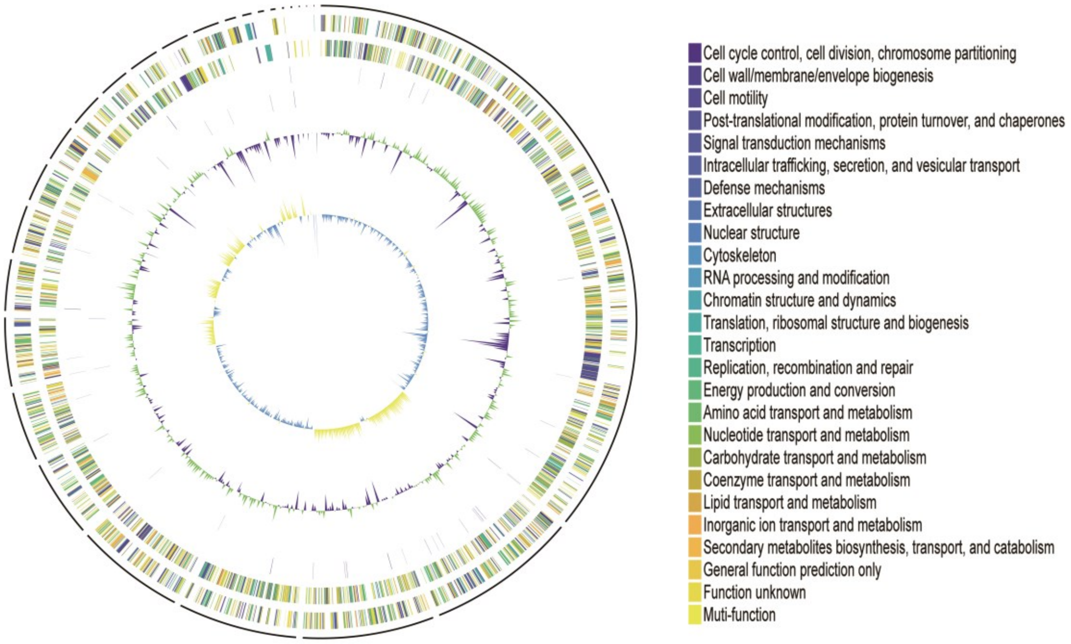

Supplement: Supplementary file 8 [file Image_1.PDF]
