# Supplementary material for: Genome Analysis of Multidrug-Resistant Shewanella algae Isolated From Human Soft Tissue Sample
Source: Front Pharmacol. 2018 Apr 26;9:419. doi: 10.3389/fphar.2018.00419 (PMC5932639; doi:10.3389/fphar.2018.00419)

Supplementary Figure S2. The distribution of the *S.algae* -specific CDSs with regard to cellular functions.

(A) The percentage distribution of the *S.algae* YHL-specific CDSs with regard to the predicted cellular functions.

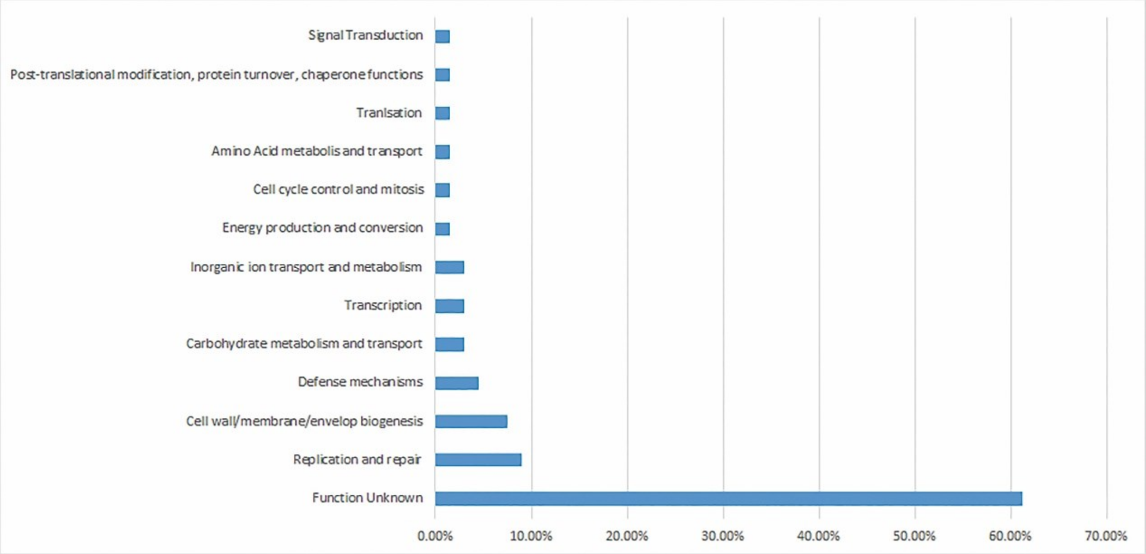

Supplement: Supplementary file 9 [file Image_2.PDF]
