# Supplementary material for: Genome Analysis of Multidrug-Resistant Shewanella algae Isolated From Human Soft Tissue Sample
Source: Front Pharmacol. 2018 Apr 26;9:419. doi: 10.3389/fphar.2018.00419 (PMC5932639; doi:10.3389/fphar.2018.00419)
Supplement: Supplementary file 10 [file Image_3.pdf]

Supplementary Figure S3. Average Nucleotide Identity (ANI) values of the S.algae YHL

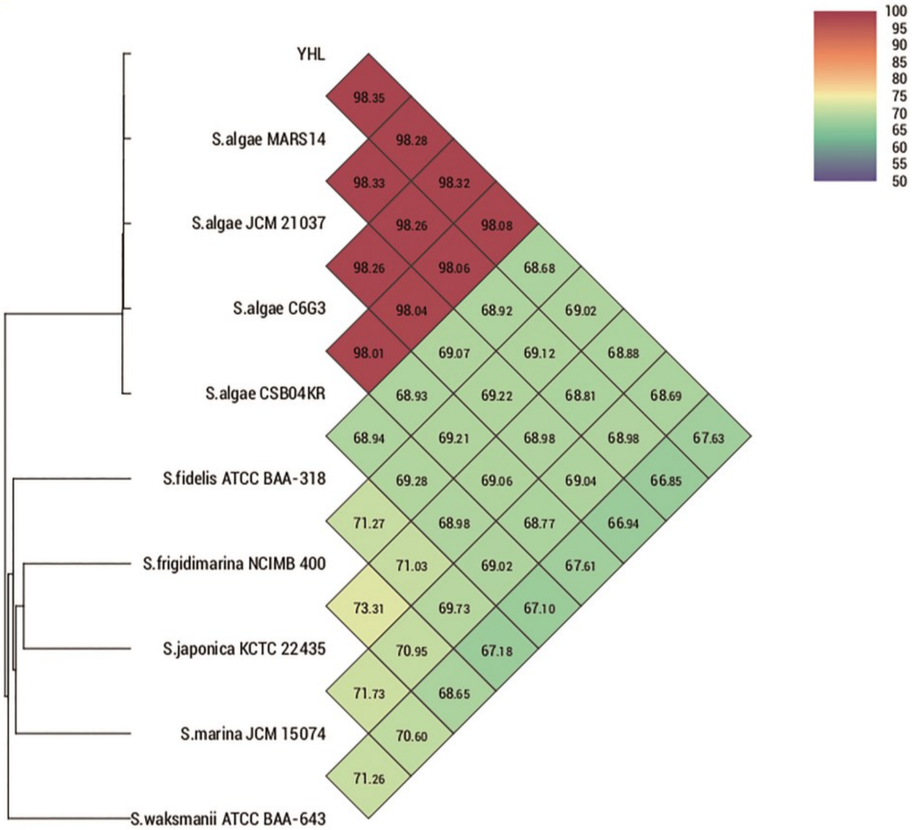

(A) The distribution between specific-CDS and shared-CDS of the S.aglae (B) The EggNOG classification of YHL-specific CDS

The Venn diagram showing of the number between specific-CDS and shared-CDS in the S.algae , and the statistics specific gene of the S.algae YHL strain

(C) Average Nucleotide Identity (ANI) values in the S.algae genome

OAT[1] uses OrthoANI to measure the overall similarity between Shewanella genome sequences.

[1] Lee, I., Kim, Y. O., Park, S. C., & Chun, J. (2015). OrthoANI: An improved algorithm and software for calculating average nucleotide identity. Int J Syst Evol Microbiol. 66: 1100-1103.
